# Urbanisation and wing asymmetry in the western honey bee (*Apis mellifera*, Linnaeus 1758) at multiple scales

**DOI:** 10.7717/peerj.5940

**Published:** 2018-12-03

**Authors:** Ryan J. Leonard, Katie K.Y. Wat, Clare McArthur, Dieter F. Hochuli

**Affiliations:** School of Life and Environmental Sciences, The University of Sydney, Camperdown, NSW, Australia

**Keywords:** Procrustes analysis, *Apis mellifera*, Urbanization, Wing asymmetry

## Abstract

Changes in the mean and variance of phenotypic traits like wing and head morphology are frequently used as indicators of environmental stress experienced during development and may serve as a convenient index of urbanization exposure. To test this claim, we collected adult western honey bee (*Apis mellifera* Linnaeus 1758, Hymenoptera, Apidae) workers from colonies located across an urbanization gradient, and quantified associations between the symmetries of both wing size and wing shape, and several landscape traits associated with urbanization. Landscape traits were assessed at two spatial scales (three km and 500 m) and included vegetation and anthropogenic land cover, total road length, road proximity and, population and dwelling density. We then used geometric morphometric techniques to determine two wing asymmetry scores—centroid size, a measure of wing size asymmetry and Procrustes distance, a measure of wing shape asymmetry. We found colony dependent differences in both wing size and shape asymmetry. Additionally, we found a negative association between wing shape asymmetry and road proximity at the three km buffer, and associations between wing shape asymmetry and road proximity, anthropogenic land cover and vegetation cover at the 500 m buffer. Whilst we were unable to account for additional variables that may influence asymmetry including temperature, pesticide presence, and parasitism our results demonstrate the potential usefulness of wing shape asymmetry for assessing the impact of certain landscape traits associated with urbanization. Furthermore, they highlight important spatial scale considerations that warrant investigation in future phenotypic studies assessing urbanization impact.

## Introduction

The unique interplay between humans and the environment in cities generates novel anthropogenic stressors such as artificial lighting, air pollution, and elevated temperatures that can profoundly affect organisms at multiple levels of biological organization (Johnson & Munshi-South, 2017; Williams, Hahs & Vesk, 2015; Alberti, Marzluff & Hunt, 2017). Such anthropogenic stressors can induce physiological responses such as cell death (Lodovici & Bigagli, 2011), and elevate stress hormone concentrations (Ouyang et al., 2015; Regoli et al., 2006). Furthermore, they may alter behaviours, including daytime activity (Dominoni & Partecke, 2015) and foraging efficiency (Gate, McNeill & Ashmore, 1995), and significantly influence the composition of biological communities, resulting in increased or decreased numbers of certain functional groups including predators and scavenging individuals (Davies, Bennie & Gaston, 2012). Given the large number of anthropogenic stressors present in cities and the magnitude with which they affect species, it is imperative we find sensitive indicators (i.e. biomarkers) that can detect detrimental effects before populations are irreversibly impacted.

Biomarkers may act as functional measures of exposure to anthropogenic stressors and environmental conditions more generally, as such, they can indicate deteriorating environmental quality and, ultimately, serve as precursors to additional physiological, behavioural, and population-level impacts (Hook, Gallagher & Batley, 2014, Hagger et al., 2006). Traditional biomarkers including abundance, reproductive output, and survival are typically time consuming and cost-prohibitive to quantify. As such, it is unsurprising urban ecologists have spent a great deal of the last two decades testing the utility of a suite of potential biomarkers including physiological and phenotypic proxies of fitness such as the quality or concentration of hormones (e.g. testosterone, Crago et al. (2011)) and organism size and mass (e.g. morphology and body size of European blackbirds, *Terdus merula* (Linnaeus, 1758, Passeriformes, Turdidae); Evans et al. (2009)).

One biomarker that shows increasing promise is fluctuating asymmetry. This type of asymmetry occurs when developmental errors resulting from exogenous environmental conditions experienced during embryogenesis produce asymmetry in morphological traits normally distributed around a mean of zero (Palmer & Strobeck, 2003). Fluctuating asymmetry occurs in bilaterally symmetrical body parts such as wings (Hoffmann, Collins & Woods, 2002; Hoffmann et al., 2005), head capsules (Leamy et al., 2015), jaws (Wiig & Bachmann, 2014), and scales (Brown et al., 2017). It is distinct from other types of asymmetry including directional asymmetry, where the population mean of the left right differences do not equal zero, and antisymmetry, where asymmetry is the norm but asymmetry towards the left and right sides are equally common, that is normally distributed around a mean of zero (Klingenberg, 2015). Fluctuating asymmetry is often considered a meaningful index of environmentally induced developmental stability (De Anna, Bonisoli-Alquati & Mousseau, 2013) and is associated with several fundamental life history components including growth, fecundity, and longevity (Møller, 1997). Additionally, fluctuating asymmetry occurs across several taxonomic boundaries including plants, vertebrates, and invertebrates (Klingenberg, 2015). Following larval exposure to certain environmental conditions under standardized conditions (i.e. laboratory), several insect taxa may exhibit increased or no change in wing size and/or wing shape fluctuating asymmetry. In the bumblebee *Bombus impatiens* (Cresson, 1863, Hymenoptera, Apidae), for example, exposure to CO_2_ increases wing shape asymmetry (Klingenberg et al., 2001), whilst low temperatures increase wing shape but not wing size asymmetry in the noctuid moth, *Helicoverpa punctigera* (Wallengren, 1860, Lepidoptera, Noctuidae), (Hoffmann, Collins & Woods, 2002). Furthermore, pollen deprivation has no impact on wing size asymmetry in neither male or female honey bees (*Apis mellifera*) (Szentgyörgyi et al., 2017).

Attempts to quantify fluctuating asymmetry in the field and associate variation with environmental conditions including gradients of contamination or urbanization have yielded mixed results (Nunes, Araújo & Marchini, 2015; Weller & Ganzhorn, 2004; Lutterschmidt, Martin & Schaefer, 2016; Banaszak-Cibicka et al., 2018). Indeed, red mason bees (*Osmia bicornis*, Linnaeus 1758, Hymenoptera, Megachilidae) collected along a zinc, cadmium, and lead contamination gradient in Poland show no difference in wing size or shape asymmetry (Szentgyörgyi et al., 2017). Similarly, euglossine bees (*Eulaema nigrita,* Lepoletier 1841, Hymenoptera, Apidae) collected from agricultural landscapes where pesticide use is prevalent did not record higher fluctuating asymmetry than individuals collected from a tropical savannah (Pinto et al., 2015). In carabid beetles, individuals display a species dependent effect of urbanization on elytra size asymmetry with ubiquitous species like *Nebria brevicollis* (Fabricuis, 1792, Coleoptera, Carabidae), and *Leistus rufomarginatus* (Duftschmid, 1812, Coleoptera, Carabidae) exhibiting no effect, yet other species including *Platynus obscurus* (Herbst, 1784, Coleoptera, Carabidae) and *Leistus ferrugineus* (Linnaeus 1758, Coleoptera, Carabidae) showing increased asymmetry with decreasing distance towards the city centre (Weller & Ganzhorn, 2004). Quantifying changes (if any) in the fluctuating asymmetry of species encountering urban areas and exposed to anthropogenic stressors of varying intensities is a critical step in determining the utility of morphometric techniques and the candidate species most appropriate for assessing asymmetry variation.

We assessed fluctuating asymmetries in wing size and wing shape, among adult western honey bees (*A. mellifera*) collected from colonies located along an urbanization gradient in the Greater Sydney Region. The western honey bee is an excellent model organism to study the effects of anthropogenic stressors and urbanization more generally due to its ubiquity across different landscape types including cities. Furthermore, wing asymmetry is common in western honey bees and frequently used to investigate the consequences of ploidy and hybridization (Smith, Crespi & Bookstein, 1997). We predicted wing shape and size fluctuating asymmetry would increase as anthropogenic stressors including anthropogenic land cover, road proximity, and housing density increased. We also sampled anthropogenic stressors at multiple spatial scales, allowing us to test how the spatial scale at which data are recorded influence associations between anthropogenic stressors and fluctuating asymmetry. We predicted the effects of anthropogenic stressors on fluctuating shape and size asymmetry would be consistent at both fine and coarse spatial scales. Multi-scale approaches like this should be a key consideration in urban ecology given they can substantially affect the interpretation of biological patterns. For example, urbanisation at fine (i.e. 200 × 200 m plots) but not coarse (i.e. 3 × 3 km) spatial scales explains variation in the fitness (i.e. survival) of *Lasiommata megera* (Linnaeus, 1767, Lepidoptera, Nymphalidae) caterpillars (Kaiser, Merckx & Van Dyck, 2016).

## Methods

### Sample collection and measurement

**Figure 1:**
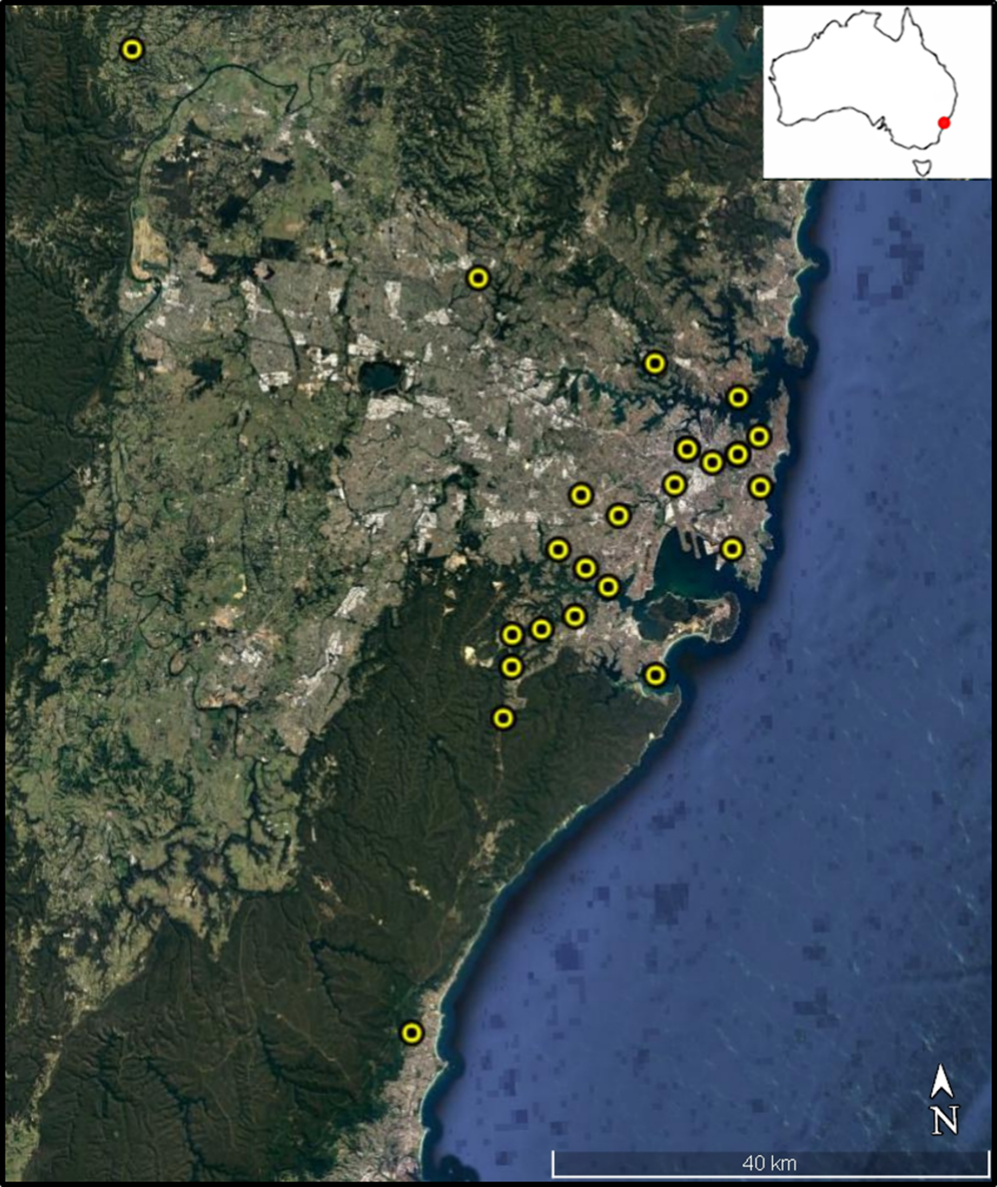
Colony sampling locations within Sydney region. Colony locations within the Greater Sydney region. Map data: Data SIO, NOAA, U.S. Navy, NGA, GEBCO. Image from Google Earth and ©2018 CNES/Airbus.

We sampled 22 permanent disease-free colonies managed by amateur bee keepers across several local government areas within the Greater Sydney Region (Fig. 1). All colonies were located within the Sydney Basin Bioregion, an area characterized by temperate climatic conditions and sandy soils derived from Hawkesbury Sandstone (NPWS, 2003). Mean annual temperature in this region is 16 °C and 1,000 mm average annual rainfall. In each case, bee keepers collected between 10 and 15 dead bees found at the colony entrance (i.e. landing board) each morning over a week-long collection period, from the 8th to the 14th of February 2016. The disposal of dead nest mates from within the colony by honey bee ‘undertakers,’ is a common behavioural response that facilitates colony hygiene (Visscher, 1983). These dead bees are typically older and have likely transitioned through all stages of the honey bee temporal caste system including cell cleaner, nurse, and forager (Langstroth, 1857). By using these bees, we attempted to reduce temporal dependent differences in exposure to environmental stressors during development. Asymmetry and, especially, fluctuating asymmetry appears during development and is believed to reflect developmental instability (Palmer, 1996). After collection by bee keepers, bees were stored in ethanol until analyses.

**Figure 2:**
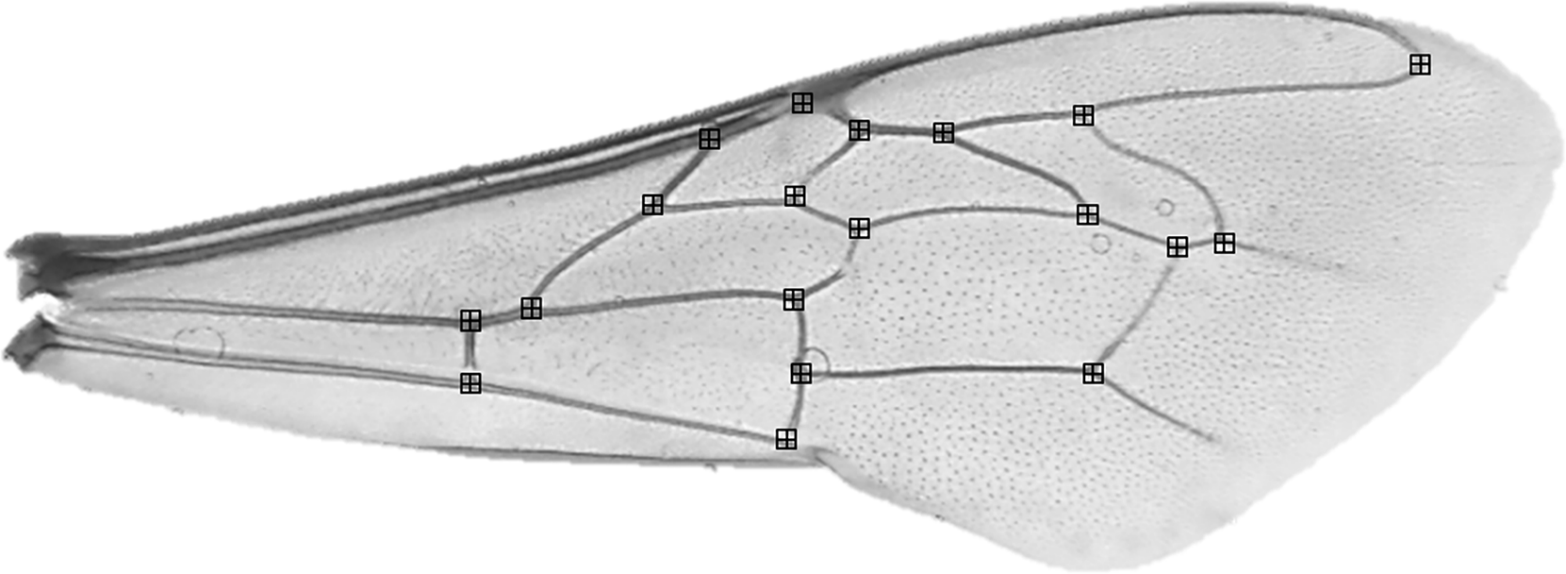
*Apis mellifera* forewing with landmark locations.

Before morphometric analyses, the forewings of 10 randomly selected individuals per colony were detached at the base of each wing, placed on a microscope slide and secured with a coverslip. Bees with damaged wings (i.e. wings where all 19 wing vein junctions were not present) were excluded. Asymmetry has been verified in small sample sizes including <10 individuals (Palmer, 1996). Each slide was photographed at 12.5 × magnification using a compound light microscope and microscope Camera Leica MC170 HD attachment. Using tPSDig software (Rohlf, 2001), we digitized photographs and collected a set of 19 landmarks positioned at wing vein junctions (Fig. 2). To reduce measurement error, this process was repeated, independently, two times and the average taken. Right wings were also mirrored to be comparable to left wings. In all cases, measurements were made by the same person.

### Quantifying size and shape variation

We assessed wing size and shape variation between colonies using Procrustes analysis, an approach that characterizes shape mathematically using a series of transformations to remove the effects of location, scale, and rotation (Klingenberg & McIntyre, 1998, Klingenberg, Barluenga & Meyer, 2002). We conducted the analysis on landmarks collected from both the left and right wing of individuals in MorphoJ software (Klingenberg, 2011). The output from this analysis includes a set of landmark coordinates containing shape information for each wing that has been corrected for size and position. To characterise wing size, we derived centroid sizes from landmark coordinates. Centroid size describes the spread of landmarks around their centre of gravity and is calculated as the square root of the sum of squared distances of each landmark from the centroid of each wing (Klingenberg, 2015). The centroid size for each wing was used to calculate wing size asymmetry by dividing the absolute difference between the left and right centroid sizes by the mean centroid size and multiplying by 100. Higher values indicate greater wing size asymmetry between the left and right wings. To calculate wing shape variation, we used wing shape asymmetry, a measure of absolute shape differences (Klingenberg, 2015). Wing shape asymmetry is calculated by taking the square root of the sum of squared differences between corresponding right and left-wing Procrustes’ coordinates. As with wing size asymmetry, higher wing shape asymmetry values indicate greater wing shape asymmetry.

### Quantifying urbanisation

We quantified habitat variables, using ArcMap (v10.3 ESRI; Redlands, CA, USA). Initially, we created a 500 m and three km buffer around each colony (Table S1). These buffer distances reflect both short- and long-distance foraging radii for western honey bees (Beekman & Ratnieks, 2000) and allowed us to investigate the effects of spatial scale on wing size and shape variation. Spatial scale (i.e. fine vs. coarse) is a key consideration in urban ecology given scale dependent effects on phenotype are common (Lowe, Wilder & Hochuli, 2014). We determined human population density, dwelling density, per cent cover of anthropogenic land use, total length of road, and total amount of vegetation per buffer area (for further details see Table S2). We also determined the distance (km) between each colony and its nearest road. These variables were measured using Geographic Information Systems (GIS) layers obtained from the New South Wales Department of Finance, Services and Innovation, Department of Environment, Climate Change and Water and the Bureau of Statistics (ABS). We derived urban population and land use statistics from the 2016 ABS census ‘Mesh Blocks’ districts, the smallest geographical unit for which census data are available.

### Statistical analyses

Given differences in bilateral traits are typically small (i.e. ≤5% of the total variation in a given trait), fluctuating asymmetry indices are especially prone to measurement error (Merila & Biorklund, 1995). For this reason, we estimated measurement error for wing size and shape asymmetry by calculating the technical error of measurement (TEM) for each bee (Jamison & Ward, 1993; Perini et al., 2005). To calculate TEM, the difference between the first and second sets of measurements are recorded, and the square root of the sum of squares of these differences is divided by the total number of measurements times two (Jamison & Ward, 1993). TEM is expressed in the same units as those used to make the original observation and smaller TEM values indicate lower measurement error (better accuracy of the investigator to perform measurement) (Perini et al., 2005). Directional asymmetry may also be a major factor influencing total asymmetry (Smith, Crespi & Bookstein, 1997). To determine if directional asymmetry was present in sampled bees, we ran separate one-sample *t*-tests using centroid size and Procrustes coordinates 1 through 38 as response variables. A Bonferroni adjusted alpha of 0.0013 was used to account for multiple comparisons. If the left right differences in these either centroid size or Procrustes coordinates 1 through 38 deviated significantly from zero, we concluded directional asymmetry was present in wing size and shape, respectively (Pinto et al., 2015).

To determine if wing size asymmetry and wing shape asymmetry differed among colony, we ran separate independent sample Kruskal–Wallis tests, using bee as the unit of replication. This test is equivalent to a one-way ANOVA and appropriate in cases where assumptions such as normality are violated (Field, 2013).

We assessed the relationship between the habitat variables collected in ArcMap and our two asymmetry measures (i.e. wing size asymmetry and wing shape asymmetry) at both 500 m and three km spatial scales using separate multiple linear regressions. To confirm our findings, we also conducted stepwise model selection by comparing the fit of models with different variables and removing those variables that did not significantly improve the fit of the model to the data (See Table S3). We used either wing size asymmetry or wing shape asymmetry as the response variable and the following predictor variables: total amount of road, nearest straight-line distance from colony to road, anthropogenic land area, vegetation area, number of dwellings, and number of people. Given predictor variables were measured on different scales, to facilitate comparison with parameter estimates we *z*-transformed all variables by first subtracting the mean of all scores from each individual score and then dividing these new values by the standard deviation of all scores (Schielzeth, 2010). We also assessed collinearity among variables by calculating variance inflation factors and generating Pearson correlation matrices. In cases where multicollinearity occurred, we removed one of two predictor variables showing high intercorrelation. Additionally, we assessed normality using Normal P–P plots and in cases where normality was violated, data were transformed. All analyses were performed using SPSS (version 22; IBM Corp., Armonk, NY, USA).

## Results

### Measurement error and directional asymmetry

Technical error of measurement was small for both wing size (mean ± S.E. = 0.047 + 0.008) and shape (mean ± S.E. = 0.00128 + 0.00003). Mean centroid size (*t*(252) = −0.55, *p* = 0.58) and Procrustes coordinates 1–38 (*p* > 0.0013 for all coordinates) did not differ significantly from zero, indicating no directional asymmetry.

### Surrounding habitat variation among colony and among colony variation

Colonies were located across an urbanization gradient that varied greatly in road extent, anthropogenic land and vegetation cover and the densities of dwellings and human inhabitants. In the three km buffer for example, road extent varied from 52,422 to 575,130 m whist anthropogenic land and vegetation cover ranged from 10 to 92% and 0 to 88% of the total buffer area, respectively. The densities of dwellings and people varied from just 3,729 dwellings to 123,553 dwellings and 10,422 people to 250,602 people, respectively. For the 500 m buffer, road extent varied from 5,031 to 29,949 m, anthropogenic land cover ranged from 29 to 100%, and vegetation cover from 0 to 55% of the total buffer area, respectively. For 500 m and three km buffers, the nearest straight line distance from colony to road was eight m and the longest 63 m. Hive significantly affected both wing size asymmetry (*H*(25) = 62.11, *p* < 0.001) and wing shape asymmetry (*H*(25) = 45.80, *p* = 0.007).

### Wing shape asymmetry

**Figure 3:**
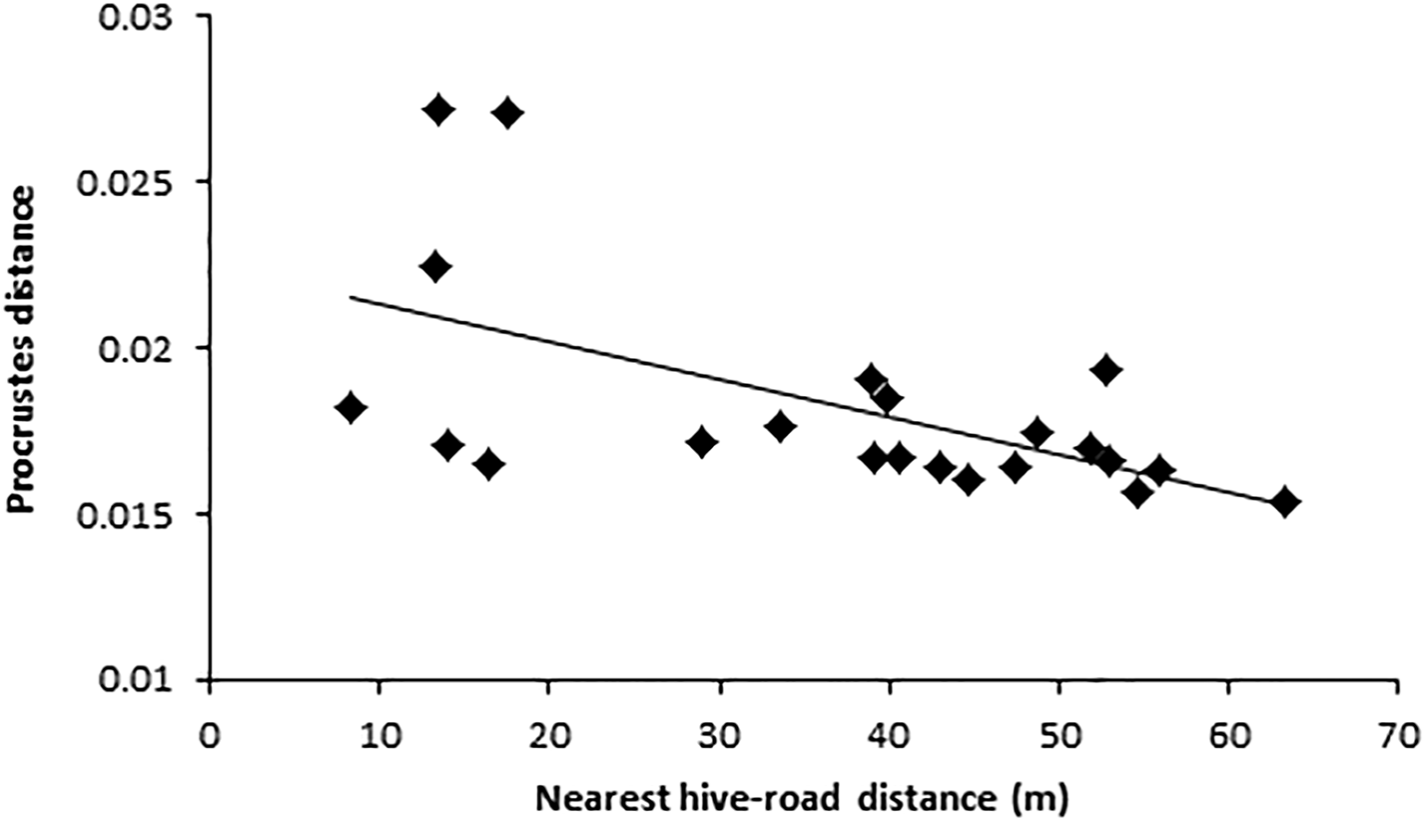
Relationship between wing shape (Procrustes distance) and nearest colony road distance. Colony-road-distance was the same in both 500 m and three km buffers. Each data point indicates the average Procrustes distance value per colony (*n* = 10 individuals). Colony were located across an urbanisation gradient and varied in their closest straight line proximity to nearby roads.

For the three km buffer, colony-road proximity, anthropogenic land area, vegetation area, and number of people significantly predicted wing shape asymmetry (*F*_(5,23)_ = 3.17, *p* = 0.04, *R*^2^ = 0.43; Table 1). However, only nearest straight-line distance from colony to road added significantly to the prediction, *p* = 0.01 (Table 1). Hives closer to roads contained bees with higher wing shape asymmetry values (Fig. 3).

**Table 1:** Parameter effect estimates.

Buffer	Explanatory term	Beta coefficient	*t*-value	*p*-value
Three km	Nearest straight line distance from hive to road	−0.60	−2.79	0.01
	Anthropogenic land area	−0.43	−1.52	0.15
	Number of people	0.05	0.15	0.88
	Vegetation area	−0.39	−1.36	0.19
500 m	Nearest straight line distance from hive to road	−0.57	−2.69	0.02
	Total road length	0.21	1.06	0.30
	Anthropogenic land area	−0.57	−2.69	0.02
	Number of dwellings	−0.29	−1.25	0.23
	Vegetation area	−0.57	−2.15	0.047

**Note:**

Parameter effect estimates (with associated *t*-statistic and *p*-values) for variables collected from three km and 500 m buffers surrounding each hive and included in multiple linear regressions analysing variation in Procrustes distance.

**Figure 4:**
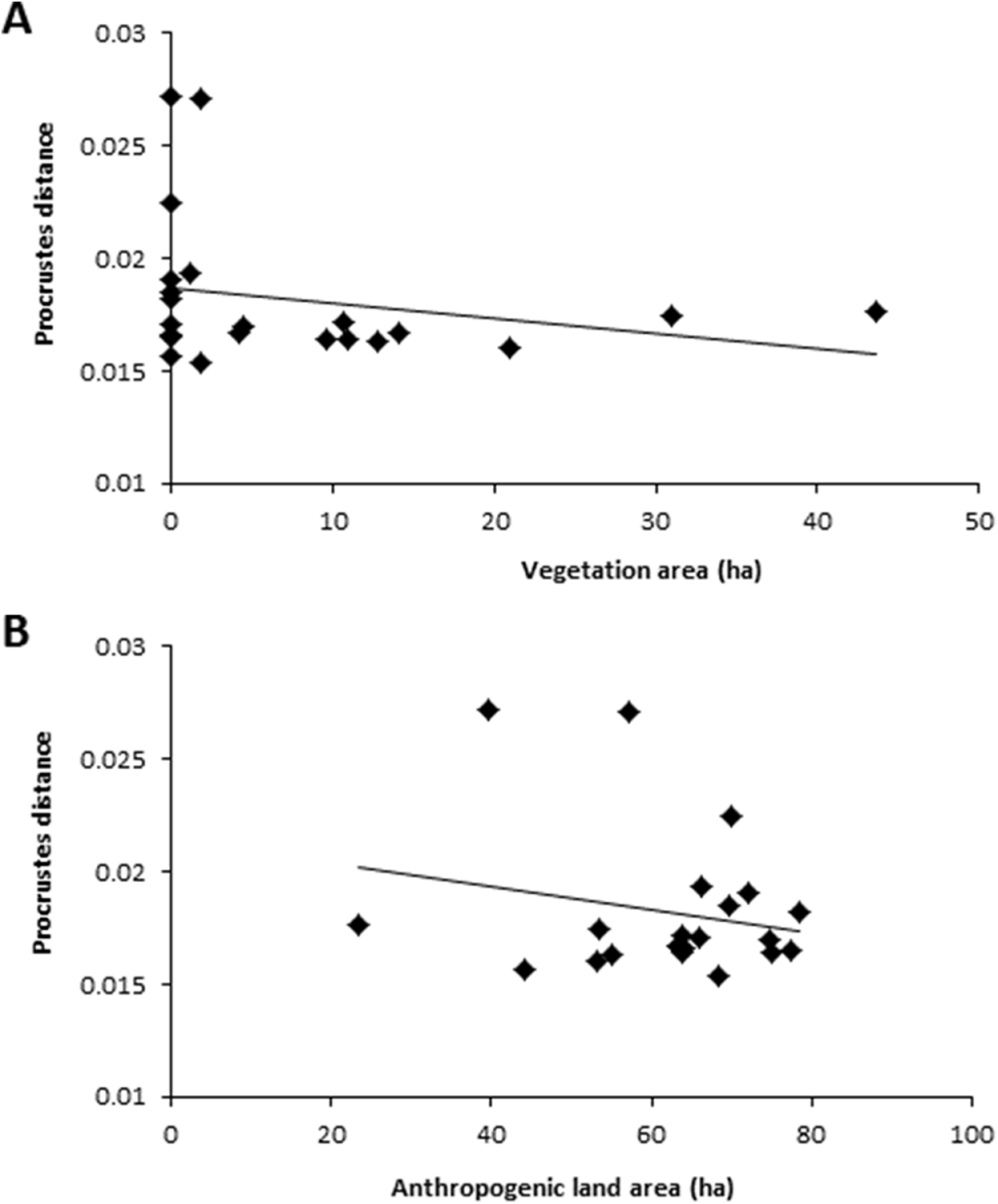
Relationship between Procrustes distance and two landscape traits, vegetation area and anthropogenic land area. (A) Relationship between Procrustes distance and vegetation area within 500 m. (B) Relationship between Procrustes distance and anthropogenic land area. Each data point is the average Procrustes distance per hive (*n* = 10 individuals). Data collected using a 500 m buffer surrounding each hive.

For the 500 m buffer, colony-road proximity, anthropogenic land area, vegetation area, total road length, and number of dwellings significantly predicted wing shape asymmetry (*F*_(5,21)_ = 4.34, *p* = 0.01, *R*^2^ = 0.58). Colony-road proximity, vegetation area, and anthropogenic land area all added statistically significantly to the prediction (Table 1). Hives closer to roads contained bees with higher wing shape asymmetry values (Fig. 3). Hives surrounded by less vegetation contained bees with higher wing shape asymmetry values (Fig. 4A), and colonies surrounded by more anthropogenic land area contained bees with lower wing shape asymmetry values (Fig. 4B).

### Wing size asymmetry

For the three km buffer, we found no effect of our predictor variables on wing size asymmetry (*F*_(4,23)_ = 1.48, *p* = 0.25, *R*^2^ = 0.26). Similarly, for the 500 m buffer, we found no effect of our predictor variables on wing size asymmetry (*F*_(5,21)_ = 1.75, *p* = 0.35, *R*^2^ = 0.18).

## Discussion

Colony location significantly affected both wing size and wing shape asymmetry. Furthermore, the effect of specific landscape features on wing shape asymmetry differed depending on the spatial scale considered. This finding is novel and highlights the important spatial scale considerations needed to assess the applicability of biomarkers including fluctuating asymmetry in the future. In our study, within the three km buffer, the only factor adding significantly to the model was colony-to-road proximity whilst, within the 500 m buffer, colony-to-road proximity and two additional factors (anthropogenic land area and vegetation area) added significantly to the model. Ultimately, our findings indicate colonies located in areas surrounded by more urban features (e.g. proximal roads, less vegetation) contain bees with higher wing shape asymmetries. Whilst these findings differ from those conducted on non-*Apis* bees (*E. nigrita*) (Pinto et al., 2015) our results are consistent with those of previous work demonstrating increased wing asymmetry in western honey bees inhabiting urban compared to less urban forested areas (Nunes, Araújo & Marchini, 2015).

Wing shape differences have previously been reported between western honey bees collected from several forest and contaminated landscape populations, including urban areas (Nunes, Araújo & Marchini, 2015). These studies grouped sites categorically as either urban or rural (Nunes, Araújo & Marchini, 2015; Silva et al., 2009). To appreciate the more nuanced effects site specific differences may have on wing asymmetry, we collected continuous measures of several landscape traits typically favoured or lost through urbanization. We suspect using continuous rather than categorical predictor variables may be more appropriate especially when the focal species is likely to respond to fine scale changes in habitat including vegetation and impervious surface. Future studies would benefit from including variables collected over fine spatial scales such as the diversity of vegetation patches directly surrounding colonies, and quality of food resources.

We found associations between road proximity and wing shape asymmetry. Similar results have been reported in the western fence lizard, *Sceloporus occidentalis* (Baird & Girard, 1852, Squamata, Phrynosomatidae), with individuals inhabiting sites containing off-highway vehicular roads reporting higher bilateral scale pattern asymmetry (Tull & Brussard, 2007). The specific mechanism(s) associated with associations between how proximal colonies were to roads and wing shape asymmetry in honey bees remains unclear. From a physiological perspective, we suspect the air pollutants occurring near roads and associated with road use (e.g. oxides of nitrogen and carbon) may be important. Carbon dioxide, a common traffic-related air pollutant, for example, significantly affects development in the moth *Manduca sexta* (Linnaeus, 1763, Lepidoptera, Sphingidae), increasing egg development time parabolically (Kerr, Phelan & Woods, 2013). Furthermore, CO_2_ exposure increases wing shape asymmetry in the bumblebee *B. impatiens* (Klingenberg et al., 2001).

Why wing asymmetry sizes and wing shape asymmetries differed between colony locations remains enigmatic, however, we suspect features of the hive and/or management practices used by bee keepers may be important. Practices increasing pesticide exposure risk including frequent spraying and/or multi-pesticide use are associated with increases in asymmetry in several bee species including the western honey bee (Abaga et al., 2011) and *Scaptotrigona* aff. *Depilis* (Moure, 1942, Hymenoptera, Apidae) (De Souza Rosa et al., 2016).

Given associations between wing shape asymmetry and vegetation and anthropogenic land area were detected at 500 m but not three km we suspect wing shape asymmetry may be resistant to changes in landscape at coarser spatial scales. This could also reflect decreased food quality and/or lower diversity and abundance of flowering plants directly surrounding each colony (i.e. within a 500 m radius). The fact wing size asymmetry was unaffected by landscape traits at either spatial scale, is unsurprising. This measure is generally constant between populations exposed to several environmental stressors including CO_2_ and cold temperatures (Hoffmann, Collins & Woods, 2002).

## Conclusion

Overall the present results indicate wing shape asymmetry in western honey bees may be related to how proximal colonies are to roads and depending on the spatial scale considered (e.g. 500 m vs. three km), other habitat variables including vegetation area and anthropogenic land area. While additional environmental conditions may also have contributed to the fluctuating asymmetry observed (e.g. temperature, pesticides, or parasitism), our findings provide support for an effect of roads. Given that roads generate light, noise, and air pollution and increase edge effects by dividing habitat, combinations of these factors should be incorporated into future observational studies. It is difficult to avoid confounding factors in such studies, however, combinations of field and manipulative laboratory studies (e.g. greenhouse reared colonies are exposed to known concentrations of traffic related air pollutants) increase the confidence with which we can establish a cause-effect relationship. One additional avenue for future work is the potential fitness consequence of the asymmetry observed. To our knowledge, this is the first study to assess the effects of spatial scale on associations between measures of wing asymmetry and landscape traits associated with urbanization. Understanding how spatial scale and the factors favoured or lost through urbanisation affect biomarkers like wing asymmetry is an important step required to quantify the utility of morphometrics for assessing urbanization impact.

## Supplemental Information

10.7717/peerj.5940/supp-1 Supplemental Information 1 Habitat data within 500 m and 3 km buffers surrounding each colony. Data were collected using GIS layers obtained from the New South Wales (NSW) Department of Finance, Services and Innovation, Department of Environment, Climate Change and Water (DECCW) and the Bureau of Statistics (ABS).

10.7717/peerj.5940/supp-2 Supplemental Information 2 Landscape traits collected in ArcMap and used in linear regression analyses. Landscape traits from each category (i.e. anthropogenic land, vegetation and road area) were summed to give a total area per buffer. This value was then used in regression analyses.

10.7717/peerj.5940/supp-3 Supplemental Information 3 Results of model selection dropping terms. In the main text we present results from multiple regressions where all landscape traits of interest are included in the multivariate model to explain variation in Procrustes distance. To test the robustness of this approach, below we also present the results of a stepwise model selection where landscape traits are removed from the model based on the *p*-value of each effect.

10.7717/peerj.5940/supp-4 Supplemental Information 4 Raw data set including landscape trait information, procrustes coordinates and centroid sizes for bees collected from hive in the Sydney region.

## Additional Information and Declarations

### Data Availability

The following information was supplied regarding data availability: The raw data are provided in a Supplemental File.
