# Supplementary material for: Urbanisation and wing asymmetry in the western honey bee (Apis mellifera, Linnaeus 1758) at multiple scales
Source: PeerJ. 2018 Dec 3;6:e5940. doi: 10.7717/peerj.5940 (PMC6282947; doi:10.7717/peerj.5940)
Supplement: Supplemental Information 1 — Data were collected using GIS layers obtained from the New South Wales (NSW) Department of Finance, Services and Innovation, Department of Environment, Climate Change and Water (DECCW) and the Bureau of Statistics (ABS). [file peerj-06-5940-s001.docx]

**Supplementary Table 1.** Habitat data within 500 m and 3 km buffers surrounding each colony. Data were collected using collected GIS layers obtained from the New South Wales (NSW) Department of Finance, Services and Innovation, Department of Environment, Climate Change and Water (DECCW) and the Bureau of Statistics (ABS).

| Buffer | Colony | Anthropogenic land cover (ha) | Vegetation cover (ha) | Road extent (m) | Colony-to-road distance (m) | Number of dwellings | Number of people |
| --- | --- | --- | --- | --- | --- | --- | --- |
| 3km | 1 | 1550.26 | 11.76 | 280332.04 | 54.69 | 59587 | 148577 |
|  | 3 | 2477.69 | 60.10 | 368870.83 | 44.76 | 50799 | 137054 |
|  | 7 | 1993.79 | 408.12 | 299289.09 | 51.89 | 34323 | 83433 |
|  | 9 | 2411.37 | 301.66 | 304744.60 | 39.89 | 24238 | 68662 |
|  | 10 | 1602.03 | 75.88 | 213738.11 | 63.41 | 21384 | 53602 |
|  | 11 | 1566.03 | 27.21 | 351768.69 | 52.93 | 65218 | 133497 |
|  | 13 | 2102.18 | 21.02 | 555394.92 | 13.39 | 113440 | 231790 |
|  | 16 | 2560.39 | 0.42 | 408559.66 | 16.46 | 64930 | 140765 |
|  | 17 | 295.00 | 2511.47 | 52422.64 | 33.53 | 3729 | 10422 |
|  | 18 | 856.13 | 1913.26 | 142967.01 | 29.06 | 11780 | 34773 |
|  | 19 | 2613.38 | 4.23 | 348410.16 | 8.33 | 50549 | 143693 |
|  | 21 | 2136.01 | 277.57 | 284483.33 | 42.97 | 43825 | 121061 |
|  | 22 | 2341.16 | 368.27 | 315621.28 | 39.22 | 41147 | 104935 |
|  | 23 | 1422.22 | 23.39 | 299250.23 | 14.04 | 62827 | 142960 |
|  | 26 | 1001.18 | 302.83 | 125284.77 | 17.56 | 18019 | 40111 |
|  | 27 | 1579.13 | 1268.28 | 244801.98 | 48.66 | 24308 | 66061 |
|  | 28 | 1983.03 | 570.28 | 264602.36 | 47.47 | 33633 | 87999 |
|  | 30 | 1035.54 | 1780.65 | 160188.24 | 40.60 | 12812 | 39387 |
|  | 31 | 1852.51 | 177.76 | 256522.33 | 55.85 | 35801 | 95756 |
|  | 32 | 1379.03 | 246.62 | 274669.79 | 52.74 | 7750 | 18410 |
|  | 33 | 2183.37 | 312.40 | 310972.96 | 38.86 | 38762 | 103269 |
|  | 34 | 2442.12 | 0.00 | 668184.95 | 13.19 | 123553 | 250602 |
| 500m | 1 | 44.08 | 0.00 | 8725.31 | 54.69 | 2332 | 5138 |
|  | 3 | 53.22 | 20.91 | 8671.95 | 44.76 | 1188 | 3134 |
|  | 7 | 74.79 | 4.51 | 8792.74 | 51.89 | 1887 | 4581 |
|  | 9 | 69.79 | 0.00 | 8087.57 | 39.89 | 958 | 2818 |
|  | 10 | 68.27 | 1.81 | 7378.94 | 63.41 | 599 | 1693 |
|  | 11 | 64.07 | 0.00 | 9087.88 | 52.93 | 1824 | 4281 |
|  | 13 | 39.56 | 0.00 | 17324.99 | 13.39 | 1896 | 3834 |
|  | 16 | 77.45 | 0.00 | 10554.82 | 16.46 | 1242 | 2733 |
|  | 17 | 23.26 | 43.69 | 5031.94 | 33.53 | 310 | 802 |
|  | 18 | 63.84 | 10.72 | 9649.91 | 29.06 | 1141 | 3462 |
|  | 19 | 78.52 | 0.00 | 11443.23 | 8.33 | 2018 | 5664 |
|  | 21 | 63.76 | 10.93 | 9297.16 | 42.97 | 1289 | 3402 |
|  | 22 | 63.36 | 4.13 | 9737.17 | 39.22 | 1015 | 2602 |
|  | 23 | 66.06 | 0.00 | 15564.57 | 14.04 | 2501 | 5891 |
|  | 26 | 57.11 | 1.79 | 7189.95 | 17.56 | 1520 | 3297 |
|  | 27 | 53.46 | 30.93 | 7264.62 | 48.66 | 795 | 2228 |
|  | 28 | 75.17 | 9.67 | 9897.37 | 47.47 | 1471 | 4206 |
|  | 30 | 63.31 | 14.11 | 9929.55 | 40.60 | 866 | 2793 |
|  | 31 | 55.14 | 12.78 | 7150.51 | 55.85 | 890 | 2649 |
|  | 32 | 66.27 | 1.18 | 19131.51 | 52.74 | 1038 | 2151 |
|  | 33 | 72.00 | 0.00 | 10374.10 | 38.86 | 1305 | 3452 |
|  | 34 | 70.07 | 0.00 | 29949.77 | 13.19 | 2008 | 5281 |
