# Supplementary material for: Urbanisation and wing asymmetry in the western honey bee (Apis mellifera, Linnaeus 1758) at multiple scales
Source: PeerJ. 2018 Dec 3;6:e5940. doi: 10.7717/peerj.5940 (PMC6282947; doi:10.7717/peerj.5940)
Supplement: Supplemental Information 2 — Landscape traits from each category (i.e. anthropogenic land, vegetation and road area) were summed to give a total area per buffer. This value was then used in regression analyses. [file peerj-06-5940-s002.docx]

Supplementary Table 2. Traits recorded in ArcMap and used to determine variables used in analyses

| Variable per buffer | Sum total of |
| --- | --- |
| Anthropogenic land area | Commercial land  Education purposed land  Hospital/medical purposed land  Industrial land  Residential land  Transport purposed land |
| Total vegetation area | Dry schlerophyll shrub grass sub-formation  Dry schlerophyll grassy sub-formation  Forested wetland  Grassy woodland  Heathland  Rainforest  Saline wetland  Wet schlerophyll shrub grass sub-formation  Wet schlerophyll grassy sub-formation |
| Total length of road | Arterial road  Distributor road  Local road  Motorway  Primary road  Urban service lane |
