# Supplementary material for: Urbanisation and wing asymmetry in the western honey bee (Apis mellifera, Linnaeus 1758) at multiple scales
Source: PeerJ. 2018 Dec 3;6:e5940. doi: 10.7717/peerj.5940 (PMC6282947; doi:10.7717/peerj.5940)
Supplement: Supplemental Information 3 — In the main text we present results from multiple regressions where all landscape traits of interest are included in the multivariate model to explain variation in Procrustes distance. To test the robustness of this approach, below we also present the results of a stepwise model selection where landscape traits are removed from the model based on the p-value of each effect. [file peerj-06-5940-s003.docx]

In the main text we present results from multiple regressions where all terms of interest are included in the multivariate model. To test the robustness of this approach, below we also present the results of a stepwise model selection where terms are removed from the model based on the p-value of each effect

Supplementary Table 3. Results of model selection dropping terms

| Buffer | Variables in model | Variable dropped | F-value | P-value |
| --- | --- | --- | --- | --- |
| 3km | Nearest straight line distance from hive to road  Anthropogenic land area  Number of people  Vegetation area | - | 3.17 | 0.04 |
|  | Nearest straight line distance from hive to road  Anthropogenic land area  Vegetation area | Number of people | 4.46 | 0.02 |
|  | Nearest straight line distance from hive to road  Anthropogenic land area | Number of people  Vegetation area | 5.04 | 0.02 |
|  | Nearest straight line distance form hive to road | Number of people  Vegetation area  Anthropogenic area | 9.96 | 0.005 |
| 500m | Nearest straight line distance from hive to road  Total road length  Anthropogenic land area  Number of dwellings  Vegetation area | - | 4.34 | 0.01 |
|  | Nearest straight line distance from hive to road  Anthropogenic land area  Number of dwellings  Vegetation area | Total road length | 5.10 | 0.007 |
|  | Nearest straight line distance from hive to road  Anthropogenic land area  Vegetation area | Total road length  Number of dwellings | 6.33 | 0.004 |
